# Will understanding vision require a wholly empirical paradigm?

**DOI:** 10.3389/fpsyg.2015.01072

**Published:** 2015-07-30

**Authors:** Dale Purves, Yaniv Morgenstern, William T. Wojtach

**Affiliations:** ^1^Duke Institute for Brain Sciences, Duke University, Durham, NC, USA; ^2^Neuroscience and Behavioral Disorders Program, Duke-NUS Graduate Medical School Singapore, Singapore, Singapore; ^3^Department of Neurobiology, Duke University, Durham, NC, USA

**Keywords:** feature detection, vision, evolution, reflex, images, empirical theory

## Abstract

Based on electrophysiological and anatomical studies, a prevalent conception is that the visual system recovers features of the world from retinal images to generate perceptions and guide behavior. This paradigm, however, is unable to explain why visual perceptions differ from physical measurements, or how behavior could routinely succeed on this basis. An alternative is that vision does not recover features of the world, but assigns perceptual qualities empirically by associating frequently occurring stimulus patterns with useful responses on the basis of survival and reproductive success. The purpose of the present article is to briefly describe this strategy of vision and the evidence for it.

## Introduction

The goal of neuroscience is to understand the operation of animal nervous systems, the human brain in particular, and in many ways this program has been remarkably successful. In one respect, however, the endeavor has been frustrated: how we perceive and successfully engage the world remains unknown. Perhaps the best example is vision. Although it is widely assumed that visual perception depends on the detection and filtering of stimulus features to correctly represent the environment, this conception is problematic. The reason is that the elements of any given image conflate its generative physical sources; thus the energy in retinal stimuli cannot specify the physical properties of objects and conditions in the world (Purves and Lotto, 2003, 2011; Purves et al., 2014). As a result, how the physiology and anatomy of the visual system enables us to succeed in the environment is deeply puzzling.

## Visual Stimuli and Perception

To appreciate this quandary, consider the objective length of a line—e.g., the edge of a ruler—and the perception it elicits. While the cause of the retinal stimulus is the ruler and its physical length, distance, and orientation in space relative to the observer, the stimulus itself depends on the ability to form an image. An eye or similar image-forming device, however, only conveys the consequences of projective geometry, not the actual length of the ruler or its other properties. Much the same reasoning applies to luminance, the most basic feature of visual stimuli. Although the intensity of light arises primarily from the combined effect of surface reflectance and illumination, as with the projection of geometric features, these and other factors giving rise to luminance values at the retina are conflated in images. As a result, luminance is no more informative about the properties of surfaces and their illumination than the projection of the ruler is about its actual length.

The inevitable loss of objective properties in retinal stimuli implies that perceptions cannot be generated simply by mapping image features back onto the world. Useful perceptions and their behavioral consequences must therefore arise in some other way.

## An Empirical Alternative

**FIGURE 1:**
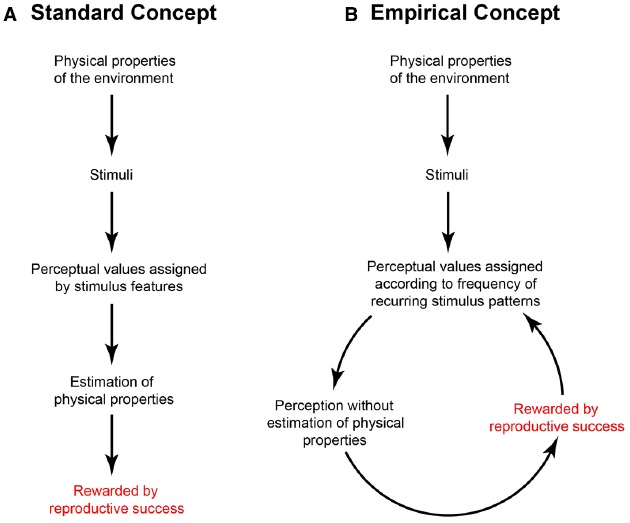
**Standard (A) and wholly empirical (B) concepts of the relationship between the physical world, sensory stimuli, perceptions, and other behaviors.** See text for explanation.

The alternative we have advocated is that trial and error responses assign perceptual values such as length or lightness according to associations that have been rewarded over evolutionary or individual time, without ever recovering or statistically estimating the properties of objects and conditions in the visual environment (Figure 1; Purves et al., 2014). Because this framework depends entirely on feedback from trial and error experience, we refer to it as “wholly empirical.”

In general terms, how such rewarded associations are implemented by evolution is well known: random changes in the organization and function of ancestral visual systems would persist—or not—according to how well the evolving system served the survival and reproductive success of the agents that harbored the variants. Any configuration of peripheral detectors and neural circuitry that associated sensory stimuli with biologically useful responses would tend to increase among the members of the population, whereas less useful associations would not. In this way the biological feedback loop in Figure 1B would progressively order the basic visual qualities we perceive (apparent length, lightness, etc.) according to their impact on biological (reproductive) success, rather than with the generative properties in the world (actual physical lengths, the reflectance and illumination values underlying luminance, etc.). The same general argument applies to lifetime learning, with the proviso that only variations in the inherited anatomical and physiological mechanisms underlying neural plasticity can be passed on, not circuit or other modifications made during life.

**FIGURE 2:**
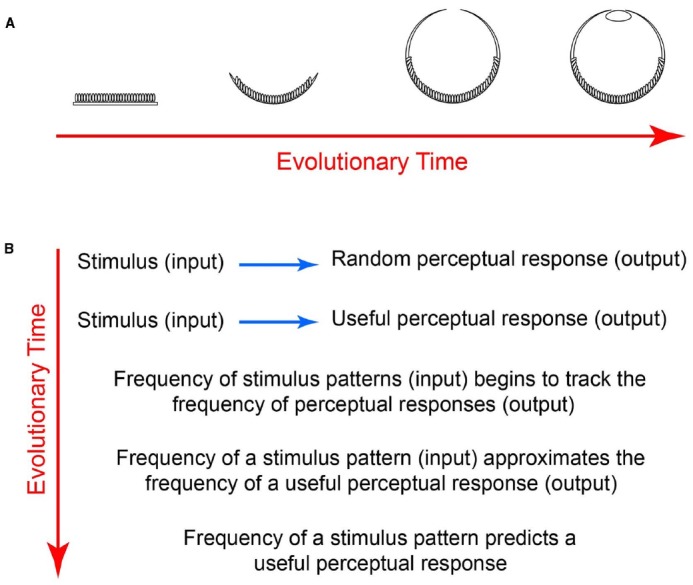
**How the frequency of occurrence of stimulus patterns comes to predict perceptions. (A)** Evolution of biological photodetectors from patches to image forming eyes. **(B)** Evolution of associations, indicating why the frequency of occurrence of rewarded stimuli eventually predicts perceptions. Early in visual evolution, different behaviorally important input patterns would be associated with responses more or less randomly. As evolution progressed, however, visual inputs would have been gradually linked to perceptual and other outputs based on survival and reproductive success. As a result, the frequency of occurrence of stimulus patterns is automatically tied to the frequency of occurrence of useful percepts.

The gradual emergence of the eye provides an example. At the outset of visual evolution, photoreceptor patches transduced radiant energy without forming images (Figure 2A). Over time, however, photoreceptor patches underwent involution to form a cup that could indicate the direction of incoming light. Further evolution then gave rise to a smaller aperture that could form low-resolution images, and eventually to lenses capable of generating high-resolution images (Land and Nilsson, 2012).

Given that reality cannot be recovered from images, however, a different strategy would need to evolve if vision were to guide behavior (Figure 2B). One way to accomplish this would be to gradually align the frequency of occurrence of stimulus input patterns with perceptual output according to behavioral feedback rather than with the properties of physical reality (Purves and Lotto, 2011; Purves et al., 2014). By doing so, the associations of input with output tends toward equivalence: every time a given pattern occurs as input, the output will more closely track the input frequency, driven by growing utility of the association. Although input-output equivalence is never fully reached in any trial and error process, after sufficient evolution (and lifetime learning), a function that describes the frequency of occurrence of input patterns should approximate the function that describes the perceptual output. Thus once evolution has reached the stage of human vision, the input-output functions are sufficiently close so that the frequency of occurrence of stimulus input patterns predicts the results of psychophysical experiments (Purves and Lotto, 2011; Purves et al., 2014).

## Evidence

**FIGURE 3:**
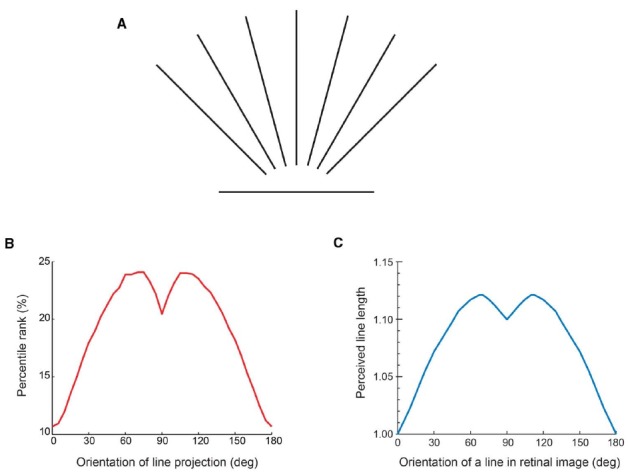
**Perceived length based on the frequency of stimulus occurrence. (A)** Lines of the same length presented in different orientations. Depending on the orientation, the line appears longer or shorter, the maximum perceived length occurring when the line stimulus is oriented approximately 30° from vertical, at which point it appears 10–15% longer than the minimum perceived length elicited by a horizontal line. **(B)** The input function of the frequency of occurrence of differently oriented 2-D line stimuli in **(A)** determined by laser range scanning natural 3-D environments. **(C)** The output function of perceived line length determined by psychophysical testing. Note the similarity of the functions in **(B)** and **(C)**, with minima arising from horizontal line projections, maxima arising from line projections oriented about 20–30° from vertical, and a dip in the functions when the projected line is vertical. (From Howe and Purves, 2005).

The evidence that visual perceptions are indeed a consequence of evolved associations between recurring input patterns and responses made on the basis of empirical reward is that the frequency of occurrence of stimulus patterns predicts perceptual experience (Purves and Lotto, 2011; Purves et al., 2014). Consider, for example, the perception of the ruler mentioned earlier. Perceptions of line length—or spatial intervals generally—has long been puzzling in that a projected line of the same measured length (e.g., the edge of the ruler) appears to have different lengths depending on its orientation (Figure 3A; Howe and Purves, 2002, 2005).

In a wholly empirical framework, the perceived length should accord with the frequency of occurrence of the lengths of projected lines in different orientations arising from the three-dimensional (3-D) geometry of the world (Figure 3B). Since physical orientation and length are conflated in two-dimensional (2-D) images, information about the actual geometry that gives rise to the image is not available to the visual system. By using the frequency of occurrence of image patterns that comprise projected length and orientation, however, this problem can be circumvented: once perception has evolved to associate the frequency of occurrence of input patterns with perceptual output, a link exists between stimulus and behavior that does not entail representing reality as such. If this explanation is correct, then the function based on the frequency of occurrence of projected lines in different orientations arising from the world (Figure 3B) should approximate the perceptual function acquired by psychophysical testing (Figure 3C), as it does. The correspondence between the functions in Figures 3B and 3C thus supports a strategy of vision in which the perception of length is determined by biological utility, as diagrammed in Figure 1B.

**FIGURE 4:**
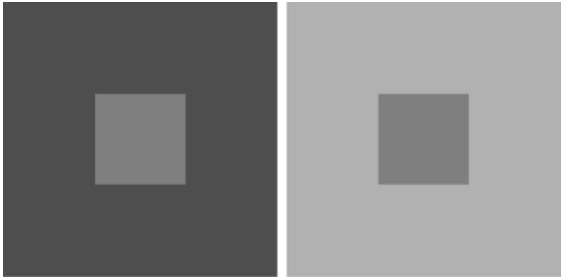
**Perceived lightness and darkness based on the frequency of stimulus occurrence.** When two targets (small squares) returning the same quantity of light to the eye are each surrounded by backgrounds (large squares) returning different quantities of light, the targets appear differently light despite having the same luminance. The frequency of occurrence of targets and contexts predicts the lightness in this example and more complex patterns as well (see Yang and Purves, 2004).

Another simple example of predicting perception on this basis is how luminance gives rise to the experience of lightness and darkness. Since luminance measures the number of photons falling on the retina, common sense suggests that measurements of light intensity and perceived lightness should be proportional. Thus if two surfaces return the same amount of light to the eye they should be perceived as equally light. In psychophysical experiments, however, this expectation routinely fails (Stevens, 1975; Purves and Lotto, 2003, 2011; Gilchrist, 2006). In the example here the central squares in Figure 4 look differently light even though their luminance is the same. The empirical explanation for this effect is much the same as the explanation of perceived length: since the light returned to the eye conflates properties of reflectance and illumination, these physical values are not available to the visual system. To circumvent this situation, vision can use the frequency of occurrence of simple patterns like these in natural scenes to rank relative lightness values according to their behavioral utility, with a function describing this input approximating a function describing the perceptual output. Thus the frequency of occurrence of the relevant stimulus patterns can predict the lightness that we actually see (Yang and Purves, 2004; Morgenstern et al., 2014a,b).

## Explaining the Discrepancy Between Objective Measures and Subjective Percepts

Relying on empirical associations to underwrite vision, however, means that perceptions are not correlated with the actual length of lines, light intensities or any other physical property, as psychophysical testing amply demonstrates. A projected line can appear longer or shorter depending on its orientation, a physical surface returning the same number of the photons to the eye can appear lighter or darker depending on the scene presented to the observer, and so on for all the other basic qualities that describe visual perception (for a review, see Purves and Lotto, 2011). Thus a consequence of this strategy is that visual perceptions will always differ from measurements of sources or images made with physical instruments such as rulers or photometers.

## Falsification of a Wholly Empirical Theory of Vision

The upshot of vision on a wholly empirical basis is that the psychophysical functions that describe the visual qualities we see can be explained pragmatically, as a way for vision to operate given the inability to recover the actual physical parameters of the world from stimuli. This conception of vision would be falsified if the frequency of occurrence of image patterns failed to predict any of the basic qualities we see, including geometrical characteristics such as lengths and angles, lightness and darkness, color, distance and motion. So far, this framework has stood up to this gamut of tests (see Purves and Lotto, 2011; Purves et al., 2014).

## Perceptions as Reflexes

Since electrophysiological evidence about the properties of neurons in the primary visual pathway and their anatomical organization in experimental animals began to emerge in the 1950s, perception has usually been conceived as the outcome of a hierarchical series of computations that entails low-level filtering and edge detection, mid-level perceptual organization (contour and shape processing), and higher-level object recognition (Marr, 1982; Riesenhuber and Poggio, 1999; Serre et al., 2007; DiCarlo et al., 2012). In the alternative framework presented here, visual perceptions are taken to be no different in kind from “simple reflexes” that evolved as rapid, automatic responses to frequently occurring stimulus patterns. In keeping with this reasoning, the human visual system generates perceptions of complex scenes in approximately the time required for retinal information to reach the visual cortex (∼80 ms) (Coppola and Purves, 1996). Together with the evidence described earlier, this observation implies that the circuitry governing perceptual and other visually-mediated responses is not dedicated to detecting, filtering, and analyzing retinal images to compute representations of the world, but to responding on the same automatic basis as any other reflex circuitry, whether in the brain or any other organ system. Concerns about the temporal plausibility of hierarchical and/or computational approaches are resolved once visual “processing” is understood as the result of activating reflex circuitry already in place, whose consequences have proven their utility over evolutionary time.

Although conceiving of visual perceptions as reflexes seems counterintuitive, there is no reason to suppose that perceptual or other behavioral responses made on an empirical basis differ from spinal or other reflexes, other than by the number of interposed neurons and synaptic connections of the input-output circuitry.

## Other Perspectives

Several other theoretical concepts of vision have also sought to move beyond the traditional idea of vision as feature detection, and we and others have reviewed these elsewhere (Gibson, 1966, 1979; Geisler, 2011; Jones and Love, 2011; Stansbury et al., 2013; Purves et al., 2014; de Wit et al., 2015). In brief, these include: (1) the use of multiple cues or viewpoints to disambiguate image uncertainty (Gibson, 1966, 1979; Freeman, 1994; de Wit et al., 2015); (2) Bayesian decision theory as a way to determine probable world-states (Brainard, 2009; Geisler, 2011; Allred and Brainard, 2013; Körding, 2014); and (3) efficient coding to reduce image redundancy (Srinivasan et al., 1982; Olshausen and Field, 1996; Schwartz and Simoncelli, 2001; Simoncelli and Olshausen, 2001). Each of these approaches has been valuable and is to some extent correct. Multiple cues and viewpoints are certainly useful in ultimately understanding what we are looking at; the use of Bayes’ theorem has provided a logical foundation of what Helmholtz referred to as unconscious inferences applied to imperfect sense data; and efficient coding has provided important insights into the receptive field structure of visual circuitry (Field, 1987; Dan et al., 1996; Olshausen and Field, 1996, 2000). But none of these approaches can explain the qualities that we actually see in response to simple stimuli like those in Figures 3 and 4, which we take to be the main challenge in seeking to rationalize biological vision.

## Summary

Visual perception is characterized by the basic qualities of lightness, brightness, color, size, distance, orientation, speed and direction of motion ordered over some range. These perceived qualities and their order within these ranges, however, do not align with reality. Understanding why requires a framework that: (1) recognizes that the major challenge for biological vision is evolving a strategy that works despite the inability of the visual system to recover physical properties; (2) shows how this challenge can be met by responses determined entirely by trial and error; (3) explains why the resulting perceptions work even though they are at odds with real-world measurements; and (4) demonstrates how and why trial and error responses eventually align perceptions with the frequency of occurrence of stimuli on the basis of rewarded associations. Although different from other interpretations of vision, this alternative framework is so far the only theory that can explain the variety of visual qualities we perceive in a principled way.

This understanding of vision and the evidence that supports it is also consistent with the receptive field properties of early level visual neurons (Hubel and Wiesel, 2005), and the goal of efficient neural coding (Graham and Field, 2007). Moreover, when evolution using these principles is simulated using neural networks, the circuitry that emerges is similar to the circuitry found in the early level visual systems of experimental animals (Ng et al., 2013; Morgenstern et al., 2014a,b). Looking forward, if vision indeed operates in this way, it is likely that other neural functions integrated with vision also operate on the basis of empirical associations.

## Author Contributions

All authors wrote and contributed to the viewpoint expressed in the manuscript.

### Conflict of Interest Statement

The authors declare that the research was conducted in the absence of any commercial or financial relationships that could be construed as a potential conflict of interest.
